# Fe-Chlorophyllin Promotes the Growth of Wheat Roots Associated with Nitric Oxide Generation

**DOI:** 10.3390/ijms11125246

**Published:** 2010-12-17

**Authors:** Min Tong, Liefeng Zhang, Yifan Wang, Hui Jiang, Yong Ren

**Affiliations:** Jiangsu Key Laboratory for Supramolecular Medicinal and Applications, College of Life Science, Nanjing Normal University, Nanjing 210046, China; E-Mails: tongmin@163.com (M.T.); yifan@gmail.com (Y.W.); Jianghui523@163.com (H.J.)

**Keywords:** Fe-chlorophyllin, nitric oxide, IAA oxidase, SOD, POD

## Abstract

Effects of Fe-chlorophyllin on the growth of wheat root were investigated in this study. We found that Fe-chlorophyllin can promote root growth. The production of nitric oxide in wheat root was detected using DAF-2DA fluorescent emission. The intensity of fluorescent in the presence of 0.1 mg/L Fe-chlorophyllin was near to that observed with the positive control of sodium nitroprusside (SNP), the nitric oxide donor. IAA oxidase activity decreased with all treatments of Fe-chlorophyllin from 0.01 to 10 mg/L. At the relatively lower Fe-chlorophyllin concentration of 0.1 mg/L, the activity of IAA oxidase displayed a remarkable decrease, being 40.1% lower than the control. Meanwhile, Fe-chlorophyllin treatment could increase the activities of reactive oxygen scavenging enzymes, such as superoxide dismutase (SOD) and peroxidase (POD), as determined using non-denaturing polyacrylamide gel electrophoresis. These results indicate that Fe-chlorophyllin contributes to the growth of wheat root associated with nitric oxide generation.

## 1. Introduction

It is well documented that various stresses lead to the overproduction of reactive oxygen species (ROS) in plants, which are highly reactive and toxic and ultimately result in oxidative stress [1]. In contrast to O_2_, these partially reduced or activated derivatives of oxygen [singlet oxygen (^1^O_2_), superoxide anion (O^2−^), hydrogen peroxide (H_2_O_2_) and hydroxyl radical (HO·)] are highly reactive and toxic, and can lead to the oxidative destruction of cells. Yu and Yang basically implies that progressive aging is associated with increasing steady-state levels of oxidatively modified biomolecules as a result of free radical reactions [2]. However, the mechanism of ROS production and its scavenging is not well known. ROS have been the uninvited companions of aerobic life [3]. Therefore, plants with the ability to scavenge and/or control the level of cellular ROS may be useful in the future to withstand various stresses.

Chlorophyllin (CHL), the sodium-copper salt and the water-soluble analogue of the ubiquitous green pigment chlorophyll, has been attributed to have several beneficial properties. Chlorophyllin, widely studied as a chlorophyll derivative, has been shown to inhibit the mutagenicity of various chemicals [4,5]. Kamat [6] reported that CHL was highly effective in protecting mitochondria, even at a low concentration of 10 μM. The antioxidant ability, at equimolar concentration, was more than that observed with ascorbic acid, glutathione, mannitol and tert-butanol. Previous reports demonstrated that commercial CHL consists of a few kinds of copper chlorophyll derivatives [7,8]. Among them, two chlorin compounds, copper chlorin e_6_ (CuCe_6_) and copper chlorin e_4_ (CuCe_4_), were suggested to be the major components responsible for the antioxidant activity of CHL [7]. This finding presented the possibility that some chlorin derivatives may be potent antioxidants. Yu *et al.* [8] reported that iron chlorin e_6_ (FeCe_6_) significantly reduced hydroxyl radical-induced thiobarbituric acid reactive substance (TBARS) formation and benzoate hydroxylation in a dose-dependent manner.

Fe-chlorophyllin is a novel hydroxyl radical scavenger in mammals [8,9], but little is known about the similar mechanisms that may operate in plants. In the present investigation we have examined the effects of Fe-chlorophyllin on the growth of wheat roots. In this study, we found that Fe-chlorophyllin could increase the activities of reactive oxygen scavenging enzyme, enhance intracellular nitric oxide generation and decrease the IAA oxidase activity in roots.

## 2. Results and Discussion

### 2.1. Fe-Chlorophyllin Promotes the Growth of Wheat Roots

**Figure 1.**
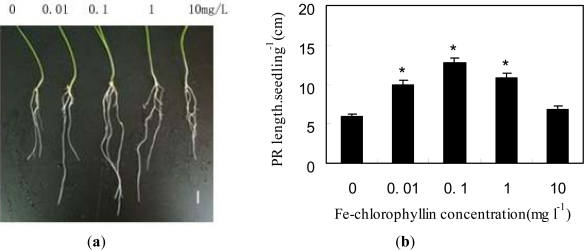
Fe-chlorophyllin regulates the root development of wheat (**a**, **b**). Seeds of wheat were soaked in distilled water containing various concentrations of Fe-chlorophyllin for 12 h and then transferred to the quarter-strength Hoagland’s nutrient solution containing different concentrations of Fe-chlorophyllin for six days after germination. (**a**) Photograph of wheat primary root formation; (**b**) The length of wheat roots. Values represent the mean of three independent experiments and vertical bars indicate standard deviations (*n* = 45 seedlings). Asterisks indicate that mean values are significantly different between the treatment and control (*p* < 0.05).

To investigate whether Fe-chlorophyllin could promote the growth of wheat roots, wheat seeds were treated with different concentrations of Fe-chlorophyllin. The results in Figure 1 show that Fe-chlorophyllin was effective in promoting the growth of wheat roots in a dose-dependent manner. For example, compared to the control alone, the addition of 0.1 mg/L Fe-chlorophyllin resulted in an increase of 2.13-fold in the length of wheat roots. However, higher concentrations of Fe-chlorophyllin contributed to inhibition of the growth of wheat roots.

Recently, Liu *et al.* [10] found that low concentration of hemin stimulated seed germination, while high concentration resulted in an inhibitory effect. Such dual effects of Fe-chlorophyllin were also confirmed in this study.

### 2.2. Changes of Anti-Oxidant Enzymes in Response to Fe-Chlorophyllin

Antioxidant enzymes can scavenge reactive oxygen species (ROS), and ROS initiate several oxidatively destructive processes. In this study, antioxidant enzymes were observed.

**Figure 2.**
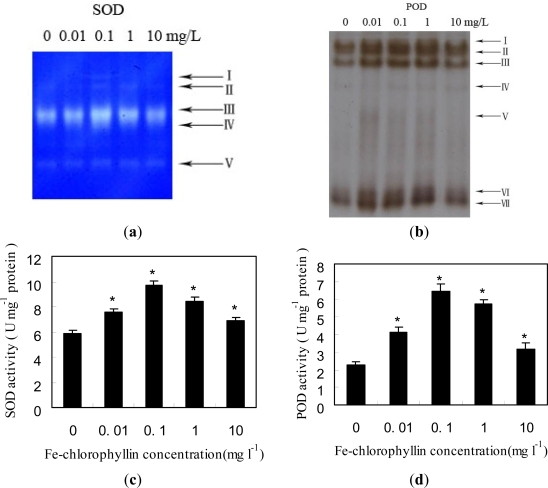
Effects of Fe-chlorophyllin on the activities of SOD and POD in the root of wheat. Seeds of wheat were soaked in the same culture solution containing 0, 0.01, 0.1, 1 and 10 mg/L Fe-chlorophyllin for 12 h and then transferred to the quarter-strength Hoagland’s nutrient solution containing different concentrations of Fe-chlorophyllin for four days after germination. After that, the treated roots homogenized, and root extracts containing 80 μg proteins were subjected to native polyacrylamide gels electrophoresis. The activities of SOD (**a**) and POD (**b**) were visualized by staining of gels (see Materials and Methods section). The arrows point to bands corresponding to isoforms. In (**c** and **d**), vertical bars represent standard deviation of the mean (*n* = 3). Asterisks indicate that mean values are significantly different between the treatment and control (*p* < 0.05).

Superoxide dismutase (SOD) activity in Fe-chlorophyllin treated root was assayed by native polyacrylamide gel electrophoresis. Five SOD isoforms were detected in the root of wheat (Figure 2a). The patterns of the five isoforms showed different responses to different Fe-chlorophyllin concentrations. Wheat treated with 0.01 mg/L and 10 mg/L did not show a significant change in activity of SOD isoforms-II, V. However, treating Fe-chlorophyllin with 0.1 mg/L and 1 mg/L caused a remarkable increase in activity of SOD isoforms-II, III, IV, and V. There is a new SOD isoform-I which only appears at the concentration 0.1 mg/L.

Seven bands of peroxidase (POD) isoforms in the root of wheat were detected (Figure 2b). All isoforms of POD appeared to show lower activity in the control, but the activities were enhanced at every Fe-chlorophyllin concentration (0.01, 0.1, 1, 10 mg/L). Activity of POD isoforms-I, II, III, IV was also stimulated with the increasing concentration of Fe-chlorophyllin up to 0.1 mg/L, where the peak activity was detected. However, activity decreased with 1 mg/L and 10 mg/L Fe-chlorophyllin.

The effects of Fe-chlorophyllin on POD and SOD activities in wheat seedling roots were investigated. As shown in Figures 2c and 2d, the activities of SOD and POD increased first, and then decreased. There were Fe-chlorophyllin concentration-dependent changes in the activities of SOD and POD. Treatments of seeds with 0.01 to 10 mg/L of Fe-chlorophyllin stimulated the activities of SOD and POD. However, at the relatively lower Fe-chlorophyllin concentration of 0.1 mg/L, the activities of SOD and POD displayed a remarkable increase, being 39.5% and 65.4% higher than the control, respectively (Figures 2c, 2d).

### 2.3. Fe-Chlorophyllin Promotes the Growth of Wheat Roots Involved in Auxin and NO Action

The positive influence of the Fe-chlorophyllin treatments on the growth of wheat root is obvious. Since nitric oxide has also been reported to be required for seedling wheat roots to develop [11], we were interested in testing the role of Fe-chlorophyllin in regulating nitric oxide generation. Recently, several studies have indicated that low levels of nitric oxide are able to mediate auxin controlled lateral root development [12,13]. Therefore, indole-3-acetic acid (IAA) oxidase and nitric oxide generation were measured in this paper.

IAA is one of the auxins that regulate plant growth and development as a plant hormone. The IAA oxidase has been suggested to play a crucial role in auxin metabolism. Evidently, the function of the enzyme is to regulate the growth of the plant by adjusting the hormonal level of IAA.

**Figure 3.**
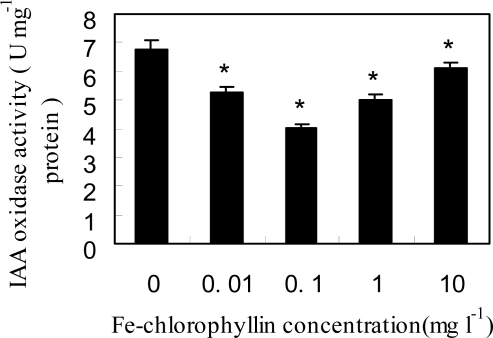
Effects of Fe-chlorophyllin on the activity of IAA oxidase in the root of wheat. Seeds of wheat were soaked in the same culture solution containing 0, 0.01, 0.1, 1 and 10 mg/L Fe-chlorophyllin for 12 h and then transferred to the quarter-strength Hoagland’s nutrient solution containing different concentrations of Fe-chlorophyllin for four days after germination. Vertical bars represent standard deviation of the mean (*n* = 3). Asterisks indicate that mean values are significantly different between the treatment and control (*p* < 0.05).

The effect of Fe-chlorophyllin on IAA oxidase activity in wheat seedling roots was investigated. As shown in Figure 3, a Fe-chlorophyllin concentration-dependent change in the activity of IAA oxidase was observed. IAA oxidase activity decreased with all treatments of Fe-chlorophyllin from 0.01 to 10 mg/L. At the relatively low Fe-chlorophyllin concentration of 0.1 mg/L, the activity of IAA oxidase was remarkably decreased, being 40.1% lower than the control.

**Figure 4.**
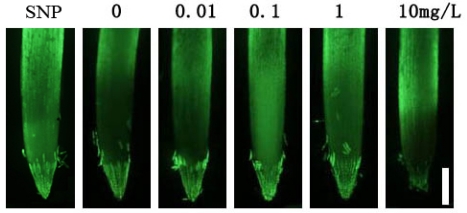
Visualization of nitric oxide generation in primary wheat roots *in vivo*. Seeds were soaked with sodium nitroprusside (SNP; 100 μmol/L) or Fe-chlorophyllin at 0, 0.01, 0.1, 1 and 10 mg/L for 12 h and then transferred to the quarter-strength Hoagland’s nutrient solution containing different concentrations of Fe-chlorophyllin for four days after germination. The seedling roots were loaded with 15 μM 4,5-diaminofluorescence (DAF-2DA) for 15 min and immediately photographed (bar = 0.5 mm).

Nitric oxide is a biologically active gaseous molecule and is proposed to be one of the important second messengers in plant cells [14]. To confirm whether Fe-chlorophyllin-regulation of root growth dependent on nitric oxide action or not, the production of nitric oxide in wheat roots was detected using DAF-2DA fluorescent emission. The control (no Fe-chlorophyllin) root displayed only very light nitric oxide staining intensity around the root apex region, while roots exposed to Fe-chlorophyllin at 0.1 mg/L stained extensively (Figure 4). The intensity of fluorescent in 0.1 mg/L was near that observed for the positive control sodium nitroprusside (SNP), the nitric oxide donor. Thus, we speculated that Fe-chlorophyllin-regulation of root growth is associated with nitric oxide generation.

**Figure 5.**
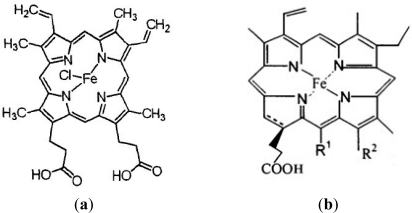
Structure of (**a**) hemin; and (**b**) Fe-chlorophyllin. R^1^ = H, CH_3_ or CH_2_COOH; R^2^ = COOH; or R^1^, R^2^ = –CH(COOH)C(O)–.

Nitric oxide plays various physiological roles in both plants and animals. Nitric oxide can enhance crop performance at any stage of its development including sowing, growth, flowering, and fruit formation or during many processes associated with the handling of the culture, such as transplantation, root formation in stem cuttings, or any other handling that could involve an oxidative stress condition for the plant. Nitric oxide might function downstream of the signal molecules of CO action by the maintenance of ion homeostasis and up-regulation of antioxidant defense in wheat seedling root tissues [15]. Hemin is the donor of CO in animals and plants [16,17]. We found that the structure of Fe-chlorophyllin and hemin are very similar, as shown in Figures 5a and 5b. We speculate that Fe-chlorophyllin, with the similar structure to hemin, could stimulate the production of the downstream signaling molecule nitric oxide. It is known that hemin is a useful plant growth regulator. It can enhance crop performance by application via soaking of seeds, spraying or irrigating [18]. The mechanism by which Fe-chlorophyllin promotes the growth of wheat roots will be further studied in our laboratory. Whether Fe-chlorophyllin is the donor of CO or not needs to be confirmed.

## 3. Experimental Section

### 3.1. Materials and Growth Conditions

Fe-chlorophyllin was obtained from the Chlorophyll Factory of Hangzhou Electrochemical Group Co., Ltd. Seeds of wheat (*Triticum aestivum* L. *Zhenmai*) were sterilized with 0.1% HgCl_2_, rinsed several times with distilled water, soaked in distilled water containing various concentrations of Fe-chlorophyllin (0, 0.01, 0.1, 1, 10 mg/L) for 12 h and germinated at 25 °C in the dark. After germination, seedlings were transferred to the quarter-strength Hoagland’s nutrient solution containing 0, 0.01, 0.1, 1, or 10 mg/L Fe-chlorophyllin, and grown at 24 ± 1 °C with 100 μmol m^−2^ s^−1^ light intensity, and a 12 h photoperiod. Treatment solutions were changed daily. When the average root length was about 5–6 cm, the roots of seedlings were harvested and immediately frozen in liquid nitrogen and stored in a −70 °C freezer for further analysis.

### 3.2. Enzyme Activity Assays

Root tissues (1.0 g) were homogenized in 10 mL of ice-cold extraction buffer (50 mM Tris-HCl, pH 7.8, 1 mM EDTA, 1 mM MgCl_2_ and 1.5% w/w polyvinylpyrrolidone). The homogenate was centrifuged at 15,000 g for 20 min at 4 °C. The supernatant was used as the crude extract to assay enzyme activities.

Analysis of guaiacol peroxidase (POD, EC 1.11.1.7) activity was based on oxidation of guaiacol using hydrogen peroxide [19]. The reaction mixture contained 2.5 mL of 50 mM potassium phosphate buffer (pH 6.1), 1 mL of 1% hydrogen peroxide, 1 mL of 1% guaiacol and 10–20 μL enzyme extract. The increase in absorbance at 420 nm was recorded. Activity was calculated using the extinction coefficient (26.6 mM^−1^ cm^−1^). One unit of POD activity was defined as the amount needed to oxidize 1 μmol guaiacol min^−1^ under the assay conditions.

Activity of superoxide dismutase (SOD, EC 1.1.5.1.1) was assayed by measuring the inhibition of photochemical reduction of nitro-blue tetrazolium (NBT) [20]. The reaction mixture (3 mL) contained 50 mM potassium phosphate buffer (pH 7.8), 10 mM methionine, 1.17 mM riboflavin and 56 mM NBT and 50 μL enzyme extract. The absorbance of the solution was measured at 560 nm. One unit of SOD was defined as the amount of enzyme causing half-maximal inhibition of the NBT reduction under the assay conditions.

### 3.3. Gel Electrophoresis

The isoenzymes of peroxidase and superoxide dismutase were separated on discontinuous polyacrylamide gels (stacking gel 5% and separating gel 10%) under non-denaturing conditions. Proteins were electrophoresed at 4 °C and 10 mA in the stacking gel followed by 15 mA in the separating gel. One gram of root tissue was homogenized with 50 mM potassium phosphate buffer (pH 7.0) including 1 mM 2-mercaptoethanol, 0.5 mM phenylmethyl, 2 mM EDTA. The homogenate was centrifuged at 15,000 g for 20 min at 4 °C. The supernatant was used for detection of isoenzymes.

Superoxide dismutase activity was determined on the gel as described by [21]. The gels were rinsed in water and incubated in the dark for 30 min at room temperature in an assay mixture containing 50 mM potassium phosphate buffer (pH 7.8), 1 mM EDTA, 0.05 mM riboflavin, 0.1 mM nitroblue tetrazolium and 0.3% *N,N,N′,N′*-tetramethylethylenediamine (TEMED). After that, the gels were rinsed with water and exposed on a light box for 10 min at room temperature until the development of colorless bands of SOD activity in a purple-stained gel were visible. For peroxidase isoforms, the gels were stained for 20 min in 0.2 M acetate buffer (pH 5.5) with 5 mM benzidine and 5 mM H_2_O_2_ [22].

### 3.4. IAA Oxidase Assay

For the IAA oxidase assay, the reaction mixture consisted of 0.66 mmol/L IAA, 1 mmol/L MnCl_2_, 0.1 mmol/L *p*-coumaric acid, 50 mmol/L Na-phosphate (pH 5.7) and enzyme extract. The reaction was monitored by following the change in absorbance at 260 nm. IAA oxidase activity was estimated as Δ*A* 260 nm min^−1^ at 25 °C [23].

### 3.5. Detection of Intracellular Nitric Oxide

Detection of root cellular nitric oxide was performed by the method described by Correa-Aragunde *et al.* [12]. Seeds of wheat were soaked in distilled water containing various concentrations of Fe-chlorophyllin. After germination, seedlings were transferred to the quarter-strength Hoagland’s nutrient solution containing various concentrations of Fe-chlorophyllin. When the average root length was about 5–6 cm, the roots of seedlings were transferred to 20 mM HEPES-NaOH (pH 7.5) buffer solution containing 15 μM specific nitric oxide fluorescent probe 4,5-diaminofluorescein diacetate (DAF-2DA). After incubation in the dark for 15 min, the roots were washed several times and immediately visualized (excitation 490 nm and emission 525 nm) using a fluorescence microscope (ECLIPSE 80i, Nikon).

### 3.6. Statistical Analysis

Each result shown in the figures is the mean of at least three replicated treatments. The significance of the differences between treatments was statistically evaluated by standard deviation and Student’s *t*-test methods.

## 4. Conclusion

Fe-chlorophyllin has antioxidant ability in mammals. In this paper, we found that it could also increase the activities of reactive oxygen scavenging enzyme, and promote the growth of wheat roots. Furthermore, Fe-chlorophyllin could enhance intracellular nitric oxide generation and decrease the IAA oxidase activity in roots. Nitric oxide is one of the important second messengers in plant cells. The function of IAA oxidase is to regulate the growth of the plant by adjusting the hormonal level of IAA. The mechanism by which Fe-chlorophyllin enhances intracellular nitric oxide needs to be further elucidated.
